# Network Reconstruction Using Nonparametric Additive ODE Models

**DOI:** 10.1371/journal.pone.0094003

**Published:** 2014-04-14

**Authors:** James Henderson, George Michailidis

**Affiliations:** Department of Statistics, University of Michigan, Ann Arbor, Michigan, United States of America; Chinese Academy of Sciences, China

## Abstract

Network representations of biological systems are widespread and reconstructing unknown networks from data is a focal problem for computational biologists. For example, the series of biochemical reactions in a metabolic pathway can be represented as a network, with nodes corresponding to metabolites and edges linking reactants to products. In a different context, regulatory relationships among genes are commonly represented as directed networks with edges pointing from influential genes to their targets. Reconstructing such networks from data is a challenging problem receiving much attention in the literature. There is a particular need for approaches tailored to time-series data and not reliant on direct intervention experiments, as the former are often more readily available. In this paper, we introduce an approach to reconstructing directed networks based on dynamic systems models. Our approach generalizes commonly used ODE models based on linear or nonlinear dynamics by extending the functional class for the functions involved from parametric to nonparametric models. Concomitantly we limit the complexity by imposing an additive structure on the estimated slope functions. Thus the submodel associated with each node is a sum of univariate functions. These univariate component functions form the basis for a novel coupling metric that we define in order to quantify the strength of proposed relationships and hence rank potential edges. We show the utility of the method by reconstructing networks using simulated data from computational models for the glycolytic pathway of *Lactocaccus Lactis* and a gene network regulating the pluripotency of mouse embryonic stem cells. For purposes of comparison, we also assess reconstruction performance using gene networks from the DREAM challenges. We compare our method to those that similarly rely on dynamic systems models and use the results to attempt to disentangle the distinct roles of linearity, sparsity, and derivative estimation.

## Introduction

### Reconstructing Biological Networks

In living organisms, biological processes such as energy metabolism or gene regulation occur through complex reaction networks involving genes, proteins, metabolites and other biochemical molecules. Understanding the mechanisms underlying these processes and the specific pathways through which they operate is of paramount interest in both basic and applied Biology, with potential applications to disease treatment and diagnosis. Part and parcel to understanding the underlying mechanisms is determining the extant relationships among genes, proteins, and metabolites. These relationships are often represented as edges in a network whose nodes represent various biochemical entities [1]. For instance, metabolism involves the conversion of one chemical into another through a series of enzyme-mediated reactions; any sequence of such reactions comprises a metabolic pathway. This pathway can be represented as a network in which the initial, final and intermediate metabolites together with other chemicals involved in the reaction sequence are represented as nodes while edges connect reactants to products. Note that in the literature, the term metabolic network is usually reserved for describing the relations among various pathways in the cell; however, we use the term as described above. In a gene regulatory network, the nodes represent genes, while edges indicate regulatory relationships. In both cases, the edges are directed to reflect the inherent asymmetry of the relationships. For such networks, there is a great deal of interest in using high-throughput data to discover relationships (edges) that can later be validated through experimental methods [2].

A number of formalisms have been proposed for learning biological networks from data [2]–[4]. Among these are: conditional independence models or Bayesian networks, direct cause-effect methodologies, and regression-style formalisms including dynamic systems models based on ordinary differential equations. As previously described, in each case nodes correspond to biochemical entities and edges to relationships among them, but the formal meaning of an edge will depend on the mathematical formalism employed.

As mentioned above, Bayesian Networks are a good model for the directed biological networks considered here. In Bayesian Networks edges correspond to statistical dependencies while nodes not connected by an edge are conditionally independent given their parents; i.e. expression levels for two genes are conditionally independent given the expression levels of their direct regulators. As another example, in a Bayesian Network for a metabolic process, the concentrations of any two metabolites might be independent given the concentrations of their direct precursors and any enzymes facilitating the formation reaction.

There are two drawbacks to conditional independence models both stemming from the fact that the network being estimated is represented as a directed acyclic graph (DAG). First, acyclic DAGs cannot accomodate biologically relevant cycles such as feedback loops [3]. Second, the number of potential DAGs grows exponentially with the number of nodes in the network necessitating approximate search strategies for even moderately sized networks [5]. While the problem of cycles can be overcome with time-series data by considering Dynamic Bayesian Networks [6], [7], this exacerbates the search problem due to the computational complexity involved for evaluating each potential network structure [5].

Nevertheless, conditional independence models are particularly well-suited for learning from gene knockout experiments. Indeed, the DREAM competitions provide strong evidence that knockouts are the most informative data type for reconstructing network topologies [8]; also see discussion in [9]. However, in practice a full suite of knockout experiments is unlikely to be available due either to expense or to some knockouts being impossible to carry out. There are a number of reasons why knockout may be infeasible; among these are lethality to the organism, and the fact that, in many cases, how to do the knockout is simply not known. Furthermore, in cases where the nodes do not represent genes, as in a metabolic pathway, there may be no logical equivalent to a knockout experiment as one cannot, say, fix the concentration of an intermediary metabolite to zero. Moreover, the most successful methods for knockout data infer direct cause effect relationships without necessarily making use of the conditional independence formalism, i.e. [10]. which tend to have difficulty distinguishing direct (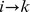) and indirect regulation (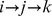) [11]. Consequently, these methods perform well when the influence and adjacency matrices are similar, but performance falls off when the adjacency matrix is much sparser than the influence or disruption matrix; i.e. when the network contains many chains of length two or more.

Network reconstruction methods utilizing time-course data have the potential to avoid some of the limitations of both conditional independence models and direct cause-effect methodologies. For the former, dynamic versions of conditional independence models can accomodate feedback loops by allowing cycles to unfold over time. For instance, reconstruction methods based on statistical time series models such as (sparse) vector autoregression [12], [13] or state space models [14] fit into this framework, although they are not usually viewed this way [5]. Time-course data can also be used to orient edges after estimating an undirected network from perturbation experiments [15]. Moreover, time-course experiments under global perturbations, including environmental stressors such as heat shock, as well as changes in initial concentrations, are generally easier to carry out than knockouts and, though requiring a greater number of measurements, have lower setup costs. Finally, time-course methods are potentially useful not only for network reconstruction, but also for predicting the response of the system to yet to be observed perturbations.

Network formalisms for time-course data are closely related to regression-style methods of network reconstruction in that both treat the latter as a feature selection problem. In the generic case, regression-based formalisms seek models that express the observations associated with each node in terms of functions of observed values on other nodes. The edges of the network are determined by the variables these functions depend on. For time-course data, the regression model often take the form of a dynamic system expressed using ordinary differential equations (ODEs),
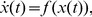
(1)where the rate of change in system components 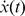 is a function, 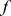, of the component trajectories, 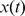. In this case, network reconstruction is a matter of finding the nonzero elements in the Jacobian 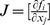. Depending on the parametric form of 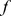, finding the nonzero elements of 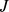 may reduce to finding nonzero parameters. A similar formulation is possible when (1) is replaced with a stochastic differential equation appropriate for single cell dynamics [16]. Though our focus is on time-course experiments, for completeness we note that nonlinear ODE models can sometimes also be fit by solving a related linear systems using data from perturbed steady states, potentially reducing the number of measurements required [17]. In the following subsection, we discuss ODE models for time-course data in greater detail.

### Differential Equation Models

Due to their long history of successful application in modeling physical phenomena, ODEs provide an attractive class of models for time-course data. There are three main decisions to be made when developing and fitting an ODE model for network reconstruction from time-course data: the model class, an approach to parameter estimation, and finally a variable selection method for the actual network reconstruction. For instance the well-known Inferelator tool employs linear models, uses a (modified) gradient-matching approach, and in its original form employs 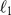 regularization for variable selection [18]–[20].

The first decision in developing an ODE model for time-course data is its parametric form. Typically the right-hand-side function (also called the slope function) is taken to be linear [21] or sigmoidal [10]. Linear ODE models are attractive because they allow one to use a number of specialized techniques, like the ability to combined multiple time-series from different experimenters [22]. While linear models also offer computational advantages and inferential simplicity, most biological processes are highly nonlinear. Nevertheless, as first-order approximations, linear models offer some protection against model misspecification. Alternatively, one can take a nonparametric approach in which the right-hand-side function is subject only to smoothness conditions. This flexible approach guards against model misspecification while allowing for nonlinearities. Other choices of models can be found in [23], though many of these have not been specifically applied to the network reconstruction task.

Approaches to estimating parameters in ODE models fall into two broad categories: trajectory matching and gradient matching. These two approaches differ in how they deal with the challenge of having equations describing the derivatives, but observations on the trajectories. The trajectory-matching approach involves choosing parameters minimizing some loss function, such as the sum of squared errors, measuring the discrepancy between a computed trajectory and the observations. If the trajectories— solutions to the initial value problem— are not available analytically, they can be found using numerical integration. The other approach, gradient-matching, instead first estimates the unobserved derivatives and then selects parameters minimizing a loss function measuring the discrepancy between the estimated derivatives and the right-hand-side function. An important feature of a gradient-matching procedure is how the derivatives are estimated. While trajectory matching is known to be statistically efficient [24] (the parameter estimates achieve a lower bound on the asymptotic variance) it can be computationally intractable for large networks. This often remains true even after one takes advantage of techniques such as differential elimination [25] for reducing the dimensionality of the system. Consequently, most ODE methods for network reconstruction employ a gradient-matching estimation scheme. Fortunately, recent statistical work shows some gradient-matching procedures are also statistically efficient [26], [27] or nearly so [28].

The final and arguably most important decision in ODE-based network reconstruction is feature (variable) selection. After all, feature selection— deciding which components should appear in each of the right-hand-side functions— ultimately determines the estimated network. Feature selection often begins with a prescreening step in which the pool of potential regulators is reduced using an information measure [19], [20]. Others choose edges using a threshold on the minimum of the objective function achieved at the parameter estimates [10]. Despite many practical successes, statistical methodology for feature selection in ODE models is an underdeveloped area. As a result, feature selection in ODE-based network reconstruction proceeds through a mixture of experience, convenience, and analogy.

Our approach, called Network Reconstruction via Dynamic Systems (NeRDS), differs from existing ODE-based methods in the following respects. To begin, we model the right-hand-side function using nonparametric, additive models which are both flexible and data-adaptive. Like other approaches, NeRDS employs a gradient-matching procedure, but differs in that the derivatives are estimated using smoothing-splines rather than finite differences. Finally, we define a novel coupling metric to measure the effect of one component on another allowing us to rank potential edges based on their estimated coupling.

The remainder of the paper proceeds as follows. The Methods section provides both an overview and details of our estimation procedure. Next, we report numerical results on *in silico* data comparing the NeRDS methodology to other ODE-based methods for network reconstruction. In the Discussion Section, we synthesize the evidence these performance comparisons provide on the distinct roles of linearity and sparsity, discuss the tradeoffs that accompany the flexibility of the method presented, and point to directions for future work.

## Methods

### Overview

Consider time-course data 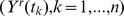 from experiments 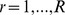. Suppose the system of interest has 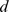 components (metabolites/genes) so that there are also 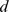 nodes in the network to be reconstructed. Thus, each 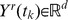 is a (random) vector of observations on these 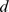 components at time 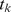. The data are taken to be noisy observations of an underlying dynamic system,
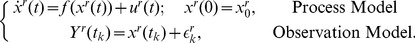
(2)The (known) inputs 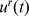 and the (possibly unknown) initial conditions 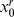 are assumed to vary across experiments so that each trajectory is independently informative of the underlying dynamics. Finally, the measurement errors 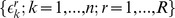 are assumed to be independent, but not (necessarily) identically distributed.

We take a nonparametric approach in which the right-hand-side function 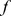 is subject only to smoothness conditions with 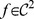 and the second derivative having bounded 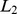 norm (see below for additional details). In contrast, other ODE-based methods treat 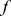 as known up to some parameters— often assuming a linear or sigmoidal function. The authors in [29] also model 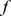 nonparametrically but their approach differs from ours in other respects. Modeling 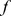 nonparametrically allows the model to adapt to arbitrary (smooth) nonlinear functions and offers robustness against model mis-specification. However, this also increases the difficulty of the estimation problem. A useful compromise for managing this trade-off is to assume that 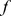 is additive so that each component is decomposed as the sum of 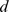 univariate functions,
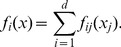
(3)Since we do not expect the network to contain edges from all 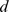 nodes to node 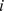, the method allows for these additive models to be sparse in the sense that for each 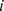 several of the 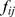 may be equivalently zero. With the additive structure in place, we can state the smoothness conditions precisely as follows. Each of the univariate functions 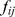 is assumed to belong to the Sobolev space 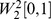 consisting of twice differentiable functions such that 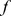 and 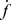 are continuous while 
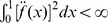
.

A second feature of the NeRDS methodology is its use of a gradient-matching approach for fitting an ODE to the data. This approach is a straightforward extension of the parametric methods in [28], [30]. One challenge in ODE estimation comes from the fact that while the trajectories are directly observed with error, only indirect information is available on the derivatives. Traditionally this has necessitated computationally expensive numerical integration at each step in the optimization procedure used for parameter estimation.

Gradient-matching approaches avoid this difficulty by first estimating the derivatives on the left-hand side of (2) and then using these plug-in estimates to simplify the parameter estimation. This not only avoids costly numerical integration, but also decouples the parameter estimation allowing each component in (2) to be learned separately. While gradient-matching approaches have a long history in applied work [23], [31], theoretical guarantees on their performance are quite recent [28].

Most ODE approaches to reconstructing regulatory networks take a gradient-matching approach in which the derivatives are estimated using finite difference approximations [18]. However, in the presence of measurement noise, derivative estimates based on finite differences are inefficient compared to smoothing-based estimates. Smoothing also allows us to estimate the entire derivative, making use of the implicit information between the observation times [28]. Moreover, the additional assumption of smoothness of the underlying trajectories (specifically the continuity of 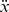) is not overly cumbersome considering the smoothness required of 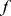, and hence of 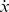, needed to ensure the existence of a unique solution to the initial value problem (2) [32]. By using smoothing splines, we improve on existing gradient-matching procedures in the network reconstruction literature by leveraging smoothness to estimate the derivatives more efficiently.

To summarize, NeRDS is a nonparametric gradient-matching procedure consisting of three stages: normalize and smooth, fit an additive ODE, and estimate coupling metrics. Details of each stage appear below.

### Details of the Estimation Procedure

**Figure 1:**
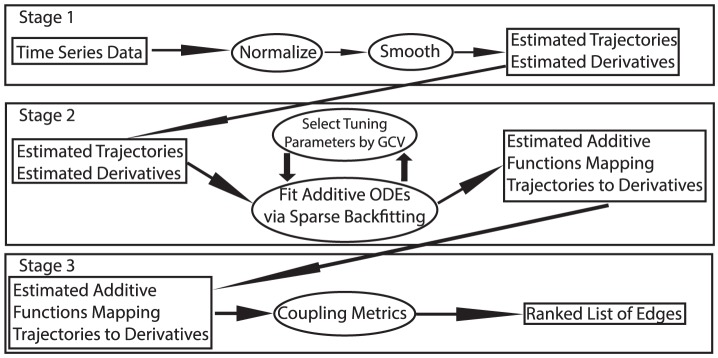
Schematic overview of the NeRDS workflow. The workflow is split into three stages. The first stage involves normalizing and smoothing the data to obtain estimates of the trajectories and derivatives. In this stage each component within each experimental run is smoothed separately. In the second stage, for each component an additive model expressing the derivative function in terms of the trajectory functions is fit using the first stage estimates. In the second stage information is combined across experiments, but the models for each component remain separate. Finally, the third stage computes pairwise couplings between components to yield a single ranked list of potential edges. Figure 2 provides a more detailed graphical overview, while the full details of each stage can be found in the Methods section.

**Figure 2:**
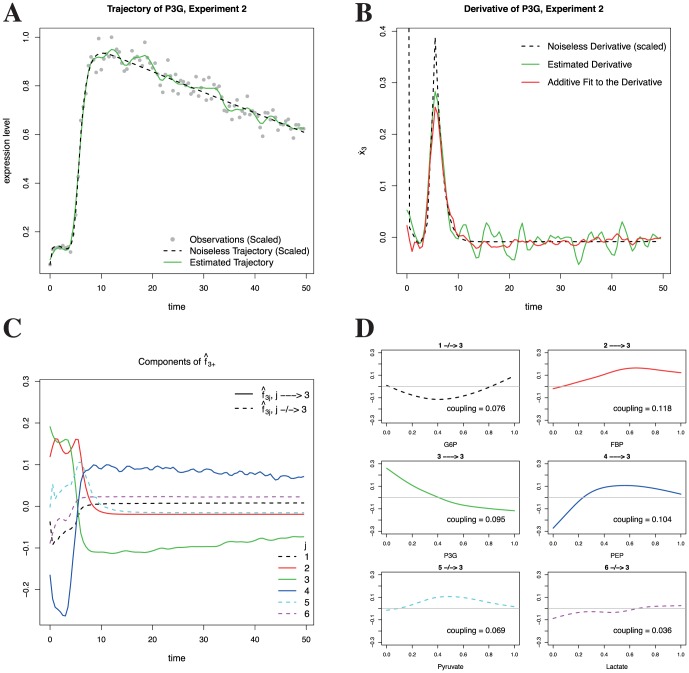
Methodological overview. This panel serves to illustrate the high-level steps in the NeRDS methodology. **Panel A** shows (simulated) data 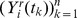 (depicted as filled circles) that are noisy measurements of the underlying trajectory (dashed black line) for P3G (i = 3) in experiment 2 (r = 2). *Step 1:* Smooth the data to obtain an estimate of the trajectory (solid green line). **Panel B** *Step 2:* Estimate the derivative (dashed black line) using the derivative of the estimated trajectory (solid green line). *Step 3:* Aggregating estimates across experiments, use backfitting to fit an additive nonparametric function (dot-dash orange line) expressing the (estimated) derivative in terms of the (estimated) trajectories. **Panel C** shows the components of the additive function, each of which is a univariate function of a single trajectory, fit to the (estimated) derivatives of P3G. Specifically shown here are 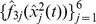 plotted against time. **Panel D** shows the component functions 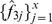 plotted over their doman (i.e. 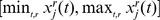). *Step 4:* Estimate the coupling using an 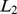 norm of the estimated component functions. In panels C and D, regulators of P3G in the underlying network are shown as solid lines, while non-regulators are shown with dashed lines.

In this section we supply details for a gradient-matching procedure for estimating the right-hand-side function 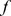 nonparametrically and using this estimate for network reconstruction. This procedure consists of three stages: 1) smoothing; 2) fitting and additive ODE; and 3) using the estimated ODE to compute the coupling between each pair of nodes. See Figure 1 for a schematic overview, Figure 2 for a graphical overview, and Algorithm S1 for a high-level description in pseudo-code.

Briefly, the three stages are as follows. In the first stage, we normalize the data and then smooth using splines to obtain estimates of the trajectories and derivatives. Normalization is done within each component across experiments, while smoothing treats each of the 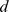 components and 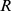 experiments separately so that there are 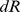 distinct smoothing problems to be solved. The second stage consists of solving 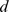 additive regression problems treating the estimated derivatives 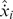 as response variables and the smoothed trajectories 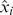 as predictors. As with other approaches based on regression, this limits attention to marginal relationships to avoid the combinatorial explosion that would otherwise occur as the number of system components 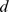 grows. Finally, in the third stage we compute the pairwise couplings using a normalized version of the 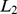 norm of the estimated functions. These couplings allow for ranking potential edges or estimating a network using a threshold. Due to the modularity of the algorithm, adjustments can be made to stage 1 to account for changes to the measurement model without subsequently affecting stages 2 or 3.

### Stage 1: Normalize and Smooth

#### Normalization

We begin by normalizing the data to ensure all components are on the same scale. In standard nonparametric modeling, it is common to scale all variables to have standard deviation one. This makes the resulting models invariant to scale and allows regularization to proceed without additional weighting schemes. However, since our observations are functional, we scale instead by the maximum observed value for each component across experiments. This serves a similar purpose and is also the approach taken in the DREAM competitions [8].

#### Using smoothing to estimate the trajectories and derivatives

The purpose of the first stage is to obtain estimated time derivatives, 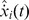; smoothed trajectory estimates 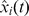 are a welcome byproduct. Beginning with the trajectories, for each component 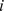 and experiment 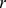 the estimated trajectories satisfy,
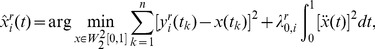
(4)where 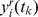 is the (normalized) observation of component 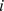 at time 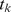 in experiment 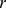 and 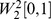 is the Sobolev space discussed in the overview. The solution is a natural cubic spline with knots at the unique time points [33]. The estimated trajectories are given by the basis function expansion 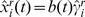 where 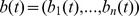 is the (row) vector of smoothing-spline basis functions evaluated at time 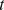 and 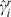 are the coefficients solving a finite-dimensional version of (4),
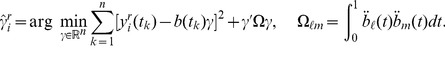
(5)Derivatives estimates are obtained by differentiating the estimated trajectories,
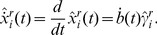
(6)Both the trajectories and derivatives are easily computed using standard software which also allows for efficient estimation of tuning parameters 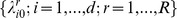 by cross validation or generalized cross validation.

### Stage 2: Fit an Additive ODE

The second stage involves finding an additive nonparametric model relating the estimated derivatives 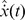 to the estimated trajectories 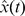. Specifically, if 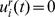 the second stage minimizes,
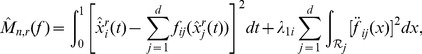
(7)so that the estimator is,
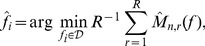
(8)with 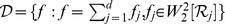 and 
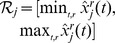
 an interval covering the estimated range of component 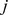 over all experiments. If 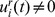 it should be subtracted from 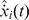 before solving the optimization problem above.

The objective function (7) is minimized using sparse backfitting [34], see Algorithm S2. Backfitting is a technique for fitting nonparametric additive models by iteratively applying univariate smoothers [35], [36]. In our case this involves first centering the estimated derivatives about the component mean and then successively solving univariate smoothing-spline problems. For instance, to update the 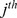 component in the 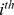 model, solve,
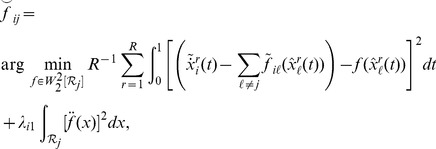
(9)where 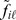 are the current estimates and 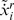 are the centered derivatives. In practice, the integrals are approximated using quadrature and the above can be accomplished by premultiplying the residual vector by a smoothing matrix 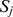. Although this is a useful simplification, rather than premultiplying by 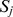 we solve the corresponding linear system using a QR decomposition to obtain updated estimates of the basis expansion coefficients 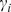. The 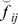 are next centered for identifiability. Finally, in order to induce sparsity, a soft-threshold is applied after solving the smoothing problem, so that the update is,
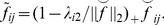
(10)


### Tuning the smoothing parameters 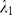 and the sparsity parameters 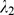


The estimators 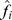 from stage two depend on tuning parameters 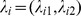. These tuning parameters depend on 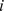 because each of the 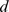 submodels is fit separately. The smoothing parameter could be allowed to vary by component, 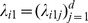, but at the cost of greatly expanding the computational cost required for tuning. In our experience this additional flexibility does not lead to significant improvement in terms of network reconstruction. The smoothing parameter 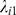 controls the roughness of the individual functions 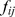 while the sparsity parameter 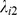 induces sparsity by setting some of the 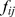 to zero. These tuning parameters are selected by minimizing the generalized cross validation score suggested by [34],
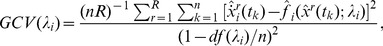
(11)where 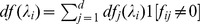 and 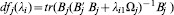 is the trace of the hat matrix projecting onto the span of the b-spline basis for the 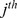 component.

While GCV allows for automatic selection of tuning parameters, overfitting— selecting 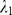 or 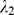 too small so the resulting model is overly complex— is always a concern. In fact, it is our experience from simulation studies that overfitting is the norm when using GCV with our methodology. As a first pass, one may choose to select 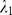 fairly large, say, 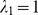, or reduce the number of knots employed, so that the resulting additive functions are nearly linear. One can then decrease 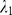 toward the value selected by GCV or increase the number of knots until an appropriate balance between flexibility and complexity is achieved, with the ‘appropriate’ balance depending on context.

Likewise, the search range for 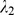 should be chosen large enough to ensure convergence of sparse backfitting in a reasonable number of iterations, yet small enough to ensure a meaningful model. Within this range GCV can serve as an objective guideline from which to justify specific departures.

Model diagnostics are an important tool for balancing complexity and flexibility. Plots overlaying estimated derivatives with linear and selected additive fits can be used to discover places where the additional flexibility is needed to achieve an adequate fit or where the complexity can be restricted without undue loss of fit. In Text S1, we illustrate use of these diagnostics for select terms from the mouse system explored in the Results section.

### Identifiability Issues

Given the complexity of the model class, it is natural to wonder about the identifiability of the additive model. To this end it is relevant to note that the smoothing matrices, 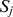 used to solve (7), are symmetric linear smoothers with eigenvalues in [0,1]. Hence, the backfitting procedure will converge to a minimizer of (7) (cf. [35], pg. 122).

However, this minimizer need not be unique despite the identifiability requirement 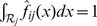. The uniqueness will depend on the *concurvity* space of the smoothers [35]. Namely, let 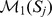 be the space spanned by the first eigenvector of 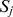. Concurvity can be thought of as the functional analog to collinearity. Then the concurvity space is,
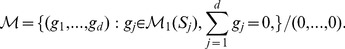
(12)If 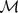 is empty then the solution to (7) will be unique. If not, the backfitting algorithm will still converge, but the solution will depend on the initial estimates of the 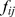.

In practice, we computationally check the identifiability of our fitted model in the following way. Since we always initialize at 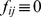, the initial estimates of the 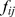 depend on the order in which the backfitting is carried out. Thus, to check for identifiability we permute this order a number of times (say 10) and compute the resulting backfitting estimators, 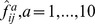. We then compute pairwise 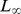 distances between the estimates,
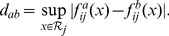
(13)When these distances are of the order of the threshold 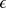 used to define convergence we take this as evidence of identifiability. Otherwise, the larger these distances the stronger the evidence against the uniqueness of each fit is. In practical terms, it also helps to overlay plots of the resulting fits and observe the extent to which they agree.

Often, when the model is not identified, it is the result of the data being insufficient for the complexity of the model fitted. Hence, reducing this complexity by increasing 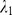 or 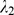 until the model becomes identified is an attractive option that we have had success with. From extensive simulation studies we find that having 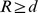, at least as many experiments as system components, generally suffices for identifiability.

### Stage 3: Coupling

The process model in (2) is specified by a set of coupled ODEs. The link between the dynamic system (2) and the target network is formalized by defining edges based on the relevant variables in the right-hand-side function 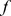. Specifically, component 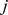 regulates component 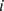 if the 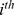 component of 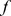 explicitly depends on 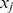,
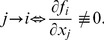
(14)


In the general case, the partial derivative 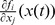 is a function of 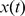
— the concentrations of all components at time 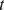. Since our working models are additive, 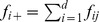, the partial derivatives 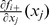 depend at most on 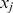. Moreover,
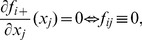
(15)allowing us to use the coupling metric,
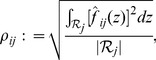
(16)with 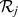 the observed range of 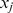 and 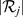 its length.

The coupling metrics are used to rank potential edges based on the strength of their regulatory influence. If desired, a single estimated network can be obtained by choosing a threshold for the coupling; only edges with coupling above this threshold are included in the estimated network. Strategies for choosing this threshold are a subject of ongoing research.

For recovering signed edges— corresponding to, say, promotion and inhibition— we define signed coupling metrics,
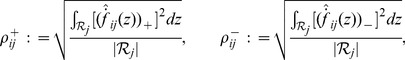
(17)by taking the positive 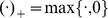 and negative 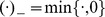 parts, respectively.

## Results

We evaluate the performance of our method *in silico* using simulated data from a variety of computational models for real biological systems. In each case, the computational model is specified by a highly nonlinear ODE with the collection of systems chosen to reflect a representative cross-section of canonical functional forms. Specifically, we choose examples from: the S-system formalism [37], sigmoidal dynamics popular with computational modelers [38], as well as the thermodynamics-based models used in the DREAM competitions [8].

Within each system, we apply the NeRDS methodology for estimating the coupling by constructing a nonparametric additive ODE and compare it to three standard parametric alternatives: linear ODEs, linear ODEs plus 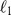 regularization (Lasso), and Inferelator 1.0 [18]. Inferelator 1.0 also employs linear ODEs and the Lasso, but takes a slightly different approach to estimation. The key differences are: 1) estimation of derivatives by finite differencing rather than smoothing splines; 2) construction of a response variable for each node combining the estimated derivative and trajectory at each time point; and 3) use of the raw observations rather than smoothed estimates of trajectories as covariates. All methods in the comparisons employ gradient-matching and use (misspecified) ODE models to estimate the network connections from time course data.

The models resulting from each method are used to rank potential edges in terms of their coupling. For the linear models, the estimated coupling is simply the appropriate coefficient in the transfer matrix. The methods' utility for network reconstruction are then compared in terms of the areas under the precision recall (AUC PR) and receiver operating characteristic (AUC ROC) curves. In sparse models the order of potential edges at the bottom of the ranked lists (corresponding to zero estimated coupling) is arbitrary. To account for this we approximate the expected AUC under random orderings of the remaining edges.

### Metabolic Pathway in Lactocaccus Lactis

**Figure 3:**
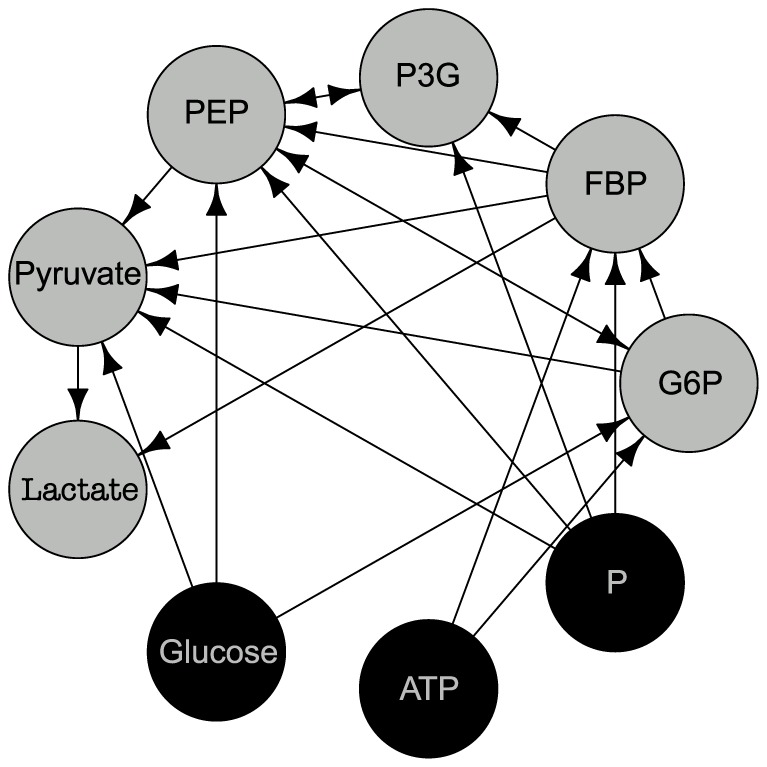
Network topology for the *Lactocaccus Lactis* system. The dark nodes with light text (glucose, ATP, and phosphorus) correspond to offline variables not explicitly modeled. We focus on reconstructing the subnetwork among the online variables (light nodes with dark text). The (simulated) data consists of vector-valued time-series of the metabolites represented by the nodes. The network, computational model and data for offline variables are taken from [39].

We begin by evaluating our methodology on an S-system developed to describe the conversion of glucose to lactate via an Embden-Meyerhof glycolytic pathway in the *Lactocaccus Lactis* bacterium [39]. The system consists of nine metabolites of which three (glucose, ATP, and phosphate) are offline variables not explicitly modeled. See Figure 3 for the network topology. For evaluation purposes we aim to reconstruct only the subnetwork among the six online variables for which the network formalism (14) makes sense.

Data for the reconstruction were obtained by simulating a suite of six experiments using the model in [39]. This suite was designed to induce curvature in the trajectories sufficient to make the nonparametric additive model identifiable. Moreover, the experiments compliment one another by ramping up the coupling among targeted subsets of edges. Specifically, this was accomplished by altering the initial abundance of each metabolite in turn,
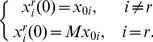
(18)The magnitude, 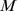, of the simulated perturbations is a simulation parameter loosely corresponding to how substantially the six experiments differ from one another.

Noiseless trajectories for each simulated experiment were computed via numerical integration. The trajectories were sampled at 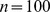 times 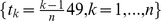 with noise added to simulate measurement error,
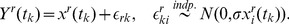
(19)


We carried out simulations for 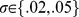 and 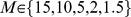 with 500 repetitions for each 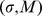 pair. The simulated data from each repetition were normalized as described in the Methods section and then used as input to four network reconstruction algorithms: 1) a NeRDS additive ODE with four interior knots, 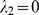 and 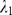 selected by GCV searching over the grid 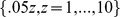; 2) a linear ODE fit by gradient matching; 3) a sparse linear ODE fit using gradient matching and lars [40]; and 4) Inferelator 1.0 [18]. All simulations were done using R [41].

Each of the first three methods utilize smoothing splines to smooth the trajectories and estimate the derivatives, as described in Methods. For the additive ODEs the sparsity parameter 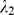 was set to 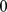 due to the small size of the system while the number of knots and search-range for 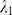 were selected by examining diagnostic plots as discussed in Methods. Moreover, following the estimation of ODE parameters using each algorithm, we ranked potential edges using the coupling metric introduced in Methods. For the three approaches employing linear ODEs, this reduces to ranking edges by the magnitude of estimated entries in the transfer matrix.

**Figure 4:**
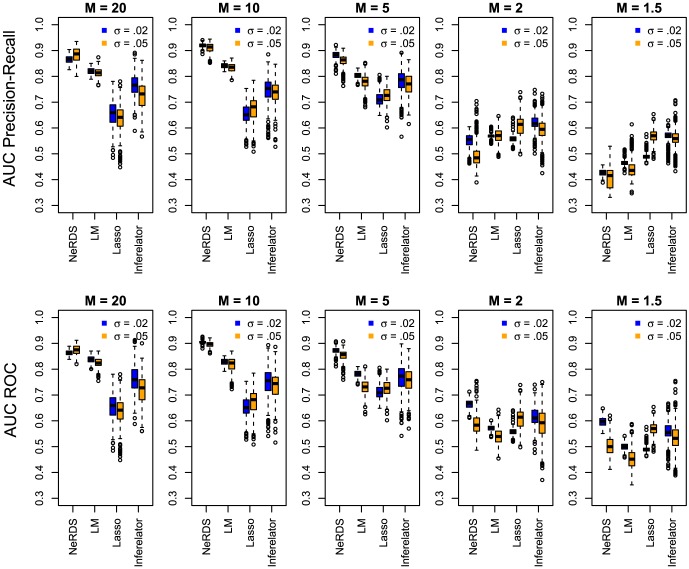
Performance evaluation on the *Lactocaccus Lactis* system. *Upper row:* Boxplots showing area under the precision-recall curves from 500 Monte Carlo simulations reconstructing the *Lactocaccus* network. *Bottom Row:* Boxplots showing area under the ROC curves. Each plot in the row corresponds to a different value of the perturbation parameter 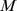 (high to low from left to right). Within each plot, boxplots are arranged according to reconstruction method; from left to right these are: additive ODEs (NeRDS), linear ODEs (LM), linear ODEs plus a Lasso penalty (Lasso), and Inferelator. For each method a pair of boxplots are presented corresponding to low noise (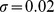, left) and moderate noise (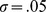, right).

The mean area under the precision-recall and ROC curves from the *Lactocaccus* simulations appear in Table 1 and Table 2, respectively. The dispersion of these measures among the 500 repetitions can be seen in the boxplots of Figure 4. Additional results using reduced sampling densities, 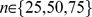, appear in Figure S3. In terms of ROC scores, the additive ODEs used by NeRDS outperformed the competitors with the exception of low-signal (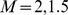) high-noise (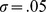) settings. Evaluated on the basis of precision-recall scores the additive ODEs performed best in high-signal settings (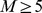), but dropped off considerably under more modest perturbations. Taken together, these results indicate that moving from linear to additive ODEs takes better advantage of sufficiently strong signals. In low-signal settings (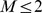) focused on precision-recall, the sparse methods, linear ODEs 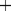 Lasso and Inferelator, outperformed the methods not utilizing sparsity.

**Table 1:** Area under the precision-recall curve for the *Lactocaccus Lactis* system.

	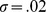	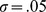
M = 15, Additive ODE	**.87** (.865,.867)	**.88** (.881, .886)
M = 15, Linear ODE	.82 (.819, .821)	.81 (.812, .815)
M = 15, Linear ODE + Lasso	.65 (.650, .659)	.64 (.632, .642)
M = 15, Inferelator 1.0	.76 (.761, .769)	.73 (.722, .731)
M = 10, Additive ODE	**.92** (.918, .920)	**.91** (.909, .912)
M = 10, Linear ODE	.84 (.840, .841)	.83 (.832, .835)
M = 10, Linear ODE + Lasso	.65 (.650, .657)	.67 (.669, .677)
M = 10, Inferelator 1.0	.75 (.741, .750)	.74 (.734, .741)
M = 5, Additive ODE	**.88** (.881, .883)	**.86** (.859, .862)
M = 5, Linear ODE	.80 (.802, .804)	.78 (.776, .781)
M = 5, Linear ODE + Lasso	.71 (.710, .715)	.73 (.723, .729)
M = 5, Inferelator 1.0	.78 (.778, .787)	.77 (.764, .772)
M = 2, Additive ODE	.55 (.549, .553)	.49 (.490, .498)
M = 2, Linear ODE	.57 (.567, .569)	.57 (.567, .572)
M = 2, Linear ODE + Lasso	.56 (.556, .559)	**.61** (.605, .612)
M = 2, Inferelator 1.0	**.62** (.618, .624)	.60 (.592, .599)
M = 1.5, Additive ODE	.43 (.426, .428)	.41 (.403, .410)
M = 1.5, Linear ODE	.47 (.464, .466)	.44 (.439, .445)
M = 1.5, Linear ODE + Lasso	.49 (.490, .493)	**.57** (.568, .572)
M = 1.5, Inferelator 1.0	**.57** (.563, .568)	.56 (.556, .562)

Performance comparison in terms of area under the precision recall curve of four methods for reconstructing the *Lactocaccus* network. The figures given are averages from 500 Monte Carlo repetitions along with confidence intervals for the mean. The parameter 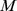 corresponds to the size of the perturbation used in generating the time series while the standard deviation of the noise is proportional to 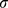. Six time series, each with 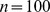 observations, are used in the reconstruction.

**Table 2:** Area under the receiver operator characteristic for the *Lactocaccus Lactis* system.

	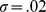	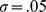
M = 15, Additive ODE	**.86** (.863, .864)	**.88** (.874, .877)
M = 15, Linear ODE	.84 (.836, .838)	.82 (.822, .825)
M = 15, Linear ODE + Lasso	.65 (.650, .659)	.64 (.632, .642)
M = 15, Inferelator 1.0	.76 (.755, .764)	.72 (.716, .727)
M = 10, Additive ODE	**.91** (.904, .906)	**.90** (.895, .897)
M = 10, Linear ODE	.83 (.826, .828)	.82 (.815, .820)
M = 10, Linear ODE + Lasso	.65 (.650, .657)	.67 (.669, .677)
M = 10, Inferelator 1.0	.75 (.744, .753)	.74 (.733, .742)
M = 5, Additive ODE	**.87** (.871, .874)	**.85** (.852, .856)
M = 5, Linear ODE	.78 (.781, .783)	.73 (.726, .731)
M = 5, Linear ODE + Lasso	.71 (.710, .715)	.73 (.723, .729)
M = 5, Inferelator 1.0	.77 (.764, .774)	.76 (.751, .759)
M = 2, Additive ODE	**.66** (.663, .666)	.59 (.584, .591)
M = 2, Linear ODE	.57 (.572, .574)	.54 (.537, .542)
M = 2, Linear ODE + Lasso	.56 (.556, .559)	**.61** (.605, .612)
M = 2, Inferelator 1.0	.61 (.612, .618)	.59 (.586, .597)
M = 1.5, Additive ODE	**.60** (.596, .599)	.50 (.499, .506)
M = 1.5, Linear ODE	.50 (.499, .502)	.45 (.450, .457)
M = 1.5, Linear ODE + Lasso	.49 (.490, .493)	**.57** (.568, .572)
M = 1.5, Inferelator 1.0	.56 (.552, .559)	.54 (.531, .540)

Performance evaluation for the *Lactocaccus* network using area under the ROC curve.

### Gene Regulatory Network in Mouse Embryonic Stem Cells

**Figure 5:**
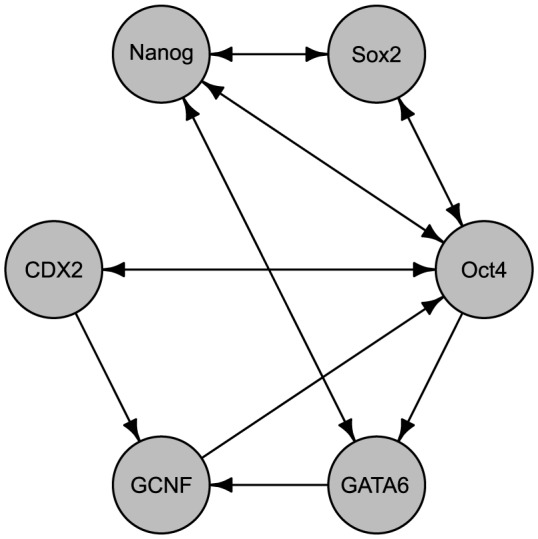
Network topology for the mouse embryonic stem cell system. This six-gene regulatory network consists of 14 edges (regulatory relationships) which we wish to discover from time-series observations of the gene expressions. The network and the computational model used to generate these observations are taken from [38].

Our second example for evaluating the NeRDS methodology is a computational model for a six-gene regulatory network developed to explain lineage determination of embryonic stem cells in mice [38]. See Figure 5 for the network topology. The system of ODEs describing the network is based on a thermodynamic model for gene regulation resulting in sigmoidal functional forms involving two- and three-way interaction terms. The setup for the simulations was nearly identical to that used for the *Lactocaccus* system described previously, with the exception that the 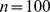 observation times 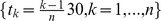 span a lesser duration. While the additive ODEs again used four interior knots and fixed 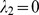, 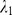 was chosen by GCV searching over the grid 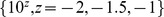. The number of knots and search range were selected to be as close to linear as possible while providing adequate fit as assessed by examining diagnostic plots from a representative dataset. See Figure S1 for an example and Text S1 for more on this point.

**Figure 6:**
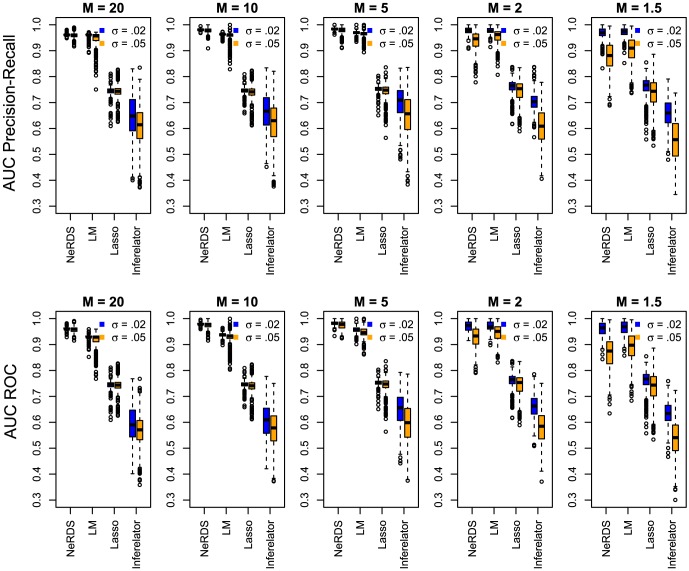
Performance evaluation on the mouse embryonic stem cell system. *Upper row:* Boxplots showing area under the precision recall curves from 500 Monte Carlo simulations reconstructing the Mouse network. *Bottom Row:* Boxplots showing area under the ROC curves. Each plot in the row corresponds to a different value of the perturbation parameter 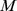 (high to low from left to right). Within each plot, boxplots are arranged according to reconstruction method; from left to right these are: additive ODEs (NeRDS), linear ODEs (LM), linear ODEs plus a lasso penalty (Lasso), and Inferelator. For each method a pair of boxplots are presented corresponding to low noise (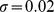, left) and moderate noise (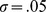, right).

Simulation results for reconstructing the mouse network appear in Table 3 and Table 4, showing mean areas under, respectively, the precision recall and ROC curves from 500 repetitions at a variety of settings. The results are also presented graphically using boxplots in Figure 6 giving a sense of each method's variability. Performance using reduced sampling densities, 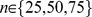, can be found in Figure S4.

**Table 3:** Area under the precision-recall curve for the mouse system.

	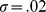	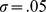
M = 15, Additive ODE	**.96** (.961, .962)	**.96** (.958, .960)
M = 15, Linear ODE	.96 (.959, .960)	.95 (.944, .948)
M = 15, Linear ODE + Lasso	.74 (.742, .746)	.74 (.739, .743)
M = 15, Inferelator 1.0	.65 (.640, .654)	.61 (.604, .618)
M = 10, Additive ODE	**.98** (.980, .981)	**.98** (.977, .978)
M = 10, Linear ODE	.96 (.963, .963)	.96 (.953, .957)
M = 10, Linear ODE + Lasso	.75 (.744, .746)	.74 (.736, .741)
M = 10, Inferelator 1.0	.66 (.655, .668)	.62 (.615, .629)
M = 5, Additive ODE	**.98** (.984, .985)	**.98** (.979, .981)
M = 5, Linear ODE	.97 (.969, .970)	.96 (.963, .965)
M = 5, Linear ODE + Lasso	.75 (.751, .753)	.74 (.740, .745)
M = 5, Inferelator 1.0	.70 (.696, .708)	.65 (.641, .656)
M = 2, Additive ODE	**.98** (.977, .979)	.94 (.935, .941)
M = 2, Linear ODE	**.98** (.976, .978)	**.96** (.953, .958)
M = 2, Linear ODE + Lasso	.76 (.758, .762)	.74 (.741, .748)
M = 2, Inferelator 1.0	.70 (.700, .707)	.61 (.601, .614)
M = 1.5, Additive ODE	.97 (.966, .970)	.88 (.873, .883)
M = 1.5, Linear ODE	**.97** (.971, .974)	**.90** (.899, .908)
M = 1.5, Linear ODE + Lasso	.76 (.757, .763)	.73 (.730, .740)
M = 1.5, Inferelator 1.0	.66 (.651, .661)	.55 (.548, .562)

Performance comparison using area under the precision-recall curve for reconstructing the mouse network.

**Table 4:** Area under the receiver operator characteristic for the mouse system.

	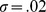	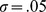
M = 15, Additive ODE	**.96** (.961, .962)	**.96** (.957, .959)
M = 15, Linear ODE	.93 (.929, .930)	.92 (.914, .919)
M = 15, Linear ODE + Lasso	.74 (.742, .746)	.74 (.739, .743)
M = 15, Inferelator 1.0	.59 (.587, .600)	.56 (.559, .570)
M = 10, Additive ODE	**.98** (.979, .980)	**.98** (.974, .976)
M = 10, Linear ODE	.94 (.936, .938)	.93 (.926, .930)
M = 10, Linear ODE + Lasso	.75 (.744, .746)	.74 (.736, .741)
M = 10, Inferelator 1.0	.60 (.598, .611)	.57 (.567, .579)
M = 5, Additive ODE	**.98** (.982, .983)	**.98** (.975, .977)
M = 5, Linear ODE	.96 (.956, .958)	.95 (.946, .949)
M = 5, Linear ODE + Lasso	.75 (.751, .753)	.74 (.740, .745)
M = 5, Inferelator 1.0	.65 (.644, .655)	.60 (.588, .602)
M = 2, Additive ODE	**.97** (.969, .972)	.93 (.925, .932)
M = 2, Linear ODE	**.97** (.968, .971)	**.95** (.943, .949)
M = 2, Linear ODE + Lasso	.76 (.758, .762)	.74 (.741, .748)
M = 2, Inferelator 1.0	.66 (.658, .665)	.58 (.577, .589)
M = 1.5, Additive ODE	**.96** (.958, .962)	.87 (.861, .872)
M = 1.5, Linear ODE	**.96** (.962, .967)	**.89** (.886, .896)
M = 1.5, Linear ODE + Lasso	.76 (.757, .763)	.73 (.730, .740)
M = 1.5, Inferelator 1.0	.63 (.630, .638)	.54 (.534, .547)

Performance comparison using area under the ROC curve for reconstructing the Mouse network.

For this network, the additive and linear ODEs were clearly the best performers overall. As with the *Lactocaccus* network, additive ODEs were the best performers in high-signal settings (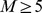). Linear ODEs had a slight advantage in low-signal (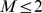) high-noise (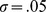) settings, while the two methods are virtually indistinguishable in low-signal (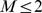), low-noise (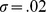) settings. Looking at the boxlots in Figure 6, we see that in low-signal (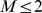), high-noise (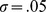) settings additive and linear ODEs both occasionally achieved perfect reconstructions, but that linear ODEs performed slightly better on average due by having higher worst-case performance.

To some extent the lack of robustness displayed may be an artifact of the simulation setting as the need to do 500 Monte Carlo repetitions precluded us from checking the stability of each model as discussed in the section Identifiability Issues above. In practice, these stability checks should suggest higher values of 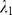 so that the additive ODESs of NeRDS become more similar to their linear counterparts. This also suggests that, all else equal, users of NeRDS should favor higher values of the smoothness parameter 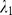 and also consider using fewer knots; see the discussion section for more on this point.

Both linear and additive ODEs outperformed Inferelator as did linear ODEs 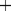 Lasso implying that this difference can not be attributed solely to sparsity. The observed differences between linear ODE 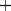 Lasso and Inferelator likely reflect the additional stability of the former due to the way in which the derivatives are estimated; in most cases, smoothing splines provide better derivative estimates than finite differencing. Nevertheless, sparsity clearly did play a role as the two methods not employing sparsity (NeRDS and linear ODEs) performed better than those that did (linear ODEs 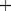 Lasso and Inferelator). Note that NeRDS did not employ sparsity because we fixed 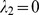.

### DREAM 3 10- and 100-Node Networks

In addition to the computational models described above, we also evaluated the NeRDS methodology on the 10- and 100-node networks from DREAM 3, challenge 4 [8], [42], [43]. This provides performance comparisons on a premier evaluation model and including the 100-node networks allows us to demonstrate that NeRDS is scalable despite its complexity relative to parametric models.

While the DREAM 3 networks represent an important point of comparison an observation is in order. Unlike the *Lactocaccus* and Mouse examples in which the time evolution of the system is fully observed, the DREAM 3 dynamics are only partially observed. This is due to the dynamic system generating the data involving unobserved proteins. The presence of unobserved variables adds an additional layer of approximation for the working models to accomodate. Including unobserved variables in the generating model has the advantage of being more faithful to the underlying science but makes *de novo* exploration more difficult. For this reason, we should not expect general exploratory models, such as linear ODEs or the additive nonparametric ODEs used by NeRDS, to perform as well as methods that take full advantage of prior scientific knowledge.

We used GeneNetWeaver [42] to generate, respectively, 10 and 100 multifactorial time series for each of the five DREAM 3 10- and 100-node networks. As discussed in [8], GeneNetWeaver generates multifactorial time series by integrating the *in silico* model from various initial conditions. These multifactorial time series are meant to simulate the networks' response to global perturbations. Here ‘global’ signifies that the targets of the perturbations are unknown, so that one can neither employ direct cause-effect methodologies, nor incorporate such cause-effect information into a dynamic model. Similar to the competition settings, these time series were generated using ODEs and adding Gaussian measurement error with standard deviation.025 to the 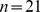 observation times on each time-series. As before, we applied each of the four methods under consideration to reconstruct these networks on the basis of these time series. We look at mean performance in terms of AUC PR and AUC ROC over 500 realizations of the measurement error for the 10-node networks, and 10 realizations of the noise for each 100-node network. The results, displayed in Table 5 and Table 6 indicate that the additive ODEs we employ compare favorably with other methods.

**Table 5:** AUC-PR and AUC-ROC for DREAM3 10-node networks.

Network	Method	AUC PR	AUC ROC
Ecoli 1	Additive ODE	0.16 (0.154, 0.163)	0.53 (0.519, 0.532)
Ecoli 1	Linear ODE	**0.20** (0.189, 0.200)	**0.60** (0.594, 0.608)
Ecoli 1	Linear ODE + Lasso	0.15 (0.150, 0.159)	0.46 (0.449, 0.461)
Ecoli 1	Inferelator 1.0	0.15 (0.146, 0.154)	0.49 (0.480, 0.494)
Ecoli 2	Additive ODE	0.20 (0.197, 0.204)	0.54 (0.537, 0.549)
Ecoli 2	Linear ODE	**0.25** (0.238, 0.253)	**0.58** (0.569, 0.583)
Ecoli 2	Linear ODE + Lasso	0.23 (0.229, 0.238)	0.50 (0.498, 0.506)
Ecoli 2	Inferelator 1.0	0.21 (0.207, 0.213)	0.52 (0.511, 0.520)
Yeast 1	Additive ODE	0.10 (0.102, 0.106)	0.45 (0.445, 0.456)
Yeast 1	Linear ODE	0.11 (0.110, 0.115)	0.45 (0.442, 0.452)
Yeast 1	Linear ODE + Lasso	0.12 (0.114, 0.119)	0.44 (0.434, 0.446)
Yeast 1	Inferelator 1.0	**0.22** (0.211, 0.220)	**0.56** (0.554, 0.565)
Yeast 2	Additive ODE	0.31 (0.307, 0.314)	0.53 (0.526, 0.536)
Yeast 2	Linear ODE	**0.36** (0.358, 0.367)	**0.59** (0.583, 0.593)
Yeast 2	Linear ODE + Lasso	0.27 (0.270, 0.278)	0.40 (0.397, 0.405)
Yeast 2	Inferelator 1.0	0.33 (0.325, 0.330)	0.45 (0.446, 0.453)
Yeast 3	Additive ODE	0.23 (0.228, 0.234)	0.48 (0.470, 0.481)
Yeast 3	Linear ODE	**0.31** (0.308, 0.319)	**0.56** (0.558, 0.571)
Yeast 3	Linear ODE + Lasso	0.28 (0.271, 0.279)	0.47 (0.463, 0.473)
Yeast 3	Inferelator 1.0	0.29 (0.290, 0.300)	0.48 (0.472, 0.484)

Performance comparisons are for a single dataset generated using GeneNetWeaver. The simulated data set contains 10 multifactorial perturbations with 21 observed time points on each. The trajectories were simulated using ODEs only. Gaussian noise with standard deviation .025 was added prior to normalization. Figures shown are means with 95% confidence intervals computed from 500 realizations of the measurement noise.

**Table 6:** AUC-PR and AUC-ROC for DREAM3 100-node networks.

Network	Method	AUC PR	AUC ROC
Ecoli 1	Additive ODE	**.109** (.106, .113)	**.639** (.627, .650)
Ecoli 1	Linear ODE	.020 (.016, .023)	.540 (.533, .547)
Ecoli 1	Linear ODE + Lasso	.022 (.018, .026)	.547 (.538, .556)
Ecoli 1	Inferelator 1.0	.067 (.059, .074)	**.622** (.611, .634)
Ecoli 2	Additive ODE	.038 (.036, .040)	**.658** (.646, .670)
Ecoli 2	Linear ODE	.021 (.014, .027)	.525 (.516, .534)
Ecoli 2	Linear ODE + Lasso	.020 (.017, .023)	.533 (.523, .543)
Ecoli 2	Inferelator 1.0	**.060**(.051, .069)	.599 (.589, .609)
Yeast 1	Additive ODE	.085 (.084, .087)	**.615** (.611, .620)
Yeast 1	Linear ODE	.053 (.047, .059)	**.609** (.601, .617)
Yeast 1	Linear ODE + Lasso	.045 (.040, .051)	.536 (.523, .549)
Yeast 1	Inferelator 1.0	**.100** (.094, .105)	.582 (.57, .594)
Yeast 2	Additive ODE	**.072** (.070, .074)	**.572** (.565, .579)
Yeast 2	Linear ODE	.048 (.048, .049)	**.568** (.563, .573)
Yeast 2	Linear ODE + Lasso	.045 (.044, .046)	.518 (.510, .526)
Yeast 2	Inferelator 1.0	.066 (.064, .067)	.517 (.513, .520)
Yeast 3	Additive ODE	.109 (.106, .111)	**.613** (.608, .619)
Yeast 3	Linear ODE	.094 (.092, .097)	**.611** (.605, .618)
Yeast 3	Linear ODE + Lasso	.089 (.087, .092)	.575 (.564, .587)
Yeast 3	Inferelator 1.0	**.118** (.115, .122)	.579 (.572, .587)

Performance comparisons are for a single dataset generated using GeneNetWeaver. The simulated data set contains 100 multifactorial perturbations with 21 observed time points on each. The trajectories were simulated using ODEs only. Gaussian noise with standard deviation .025 was added prior to normalization. The top performer(s) in each column are bolded. Figures shown are means with 95% confidence intervals computed from 10 realizations of the measurement noise.

For the additive ODEs on the 100-node networks, we first computed GCV over a range of 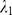 and 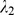 values for a single repetition from the Ecoli1 100-node network with knots at all unique data points. To improve stability and limit complexity, we fixed 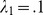 and 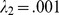 across all nodes in the 100-node networks because these values most frequently minimized GCV on the network examined. This has the effect of eliminating variability due to tuning parameter selection. For the 10-node networks, we used 4 knots and fixed 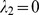, but allowed 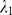 to be selected by GCV from the sequence 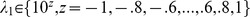.

Unlike the DREAM 3 competition, in the comparison just discussed we did not assume access to any knockdown or knockout data in accordance with our goal of improving methodology for time-course data. However, for the sake of completeness, we also provide performance comparisons on the actual data from challenge 4 of the DREAM 3 competition. Due to the small number of time series available (4 and 46, respectively, for the 10- and 100-node networks), methods not utilizing the knockout data— known to be most informative [8]— will not be competitive. For a fair comparison, we first used the knockout data to estimate an influence matrix for each network. Using this estimated influence matrix, we limited the pool of potential regulators for each submodel when fitting additive ODEs to the time series data. In summary, we screened potential regulators using the knockout experiments, and then ranked those remaining in terms of the estimated coupling.

Our approach to estimating the influence matrix was similar to that used by the top performers in the competition for estimating the first batch of edges [10]. Briefly, the idea is to use t-tests to determine which genes in a particular knockout strain have expression levels significantly different from wild-type expression. The t-tests rely on a pooled estimate of the standard deviation of the measurement noise as well as estimates of the mean wild-type expression for each gene. To improve the power of the tests, one iterates between estimating the downstream effects of each knockout and updating the estimates of the means and standard deviation based until the influence matrix is left unchanged. Means are initialized to the wild-type observations and the standard deviation is initially based on all but the direct targets of each knockout. After each update of the influence matrix, the means and standard deviation were updated using the observations estimated to be unaffected by the knockouts.

Estimating the influence matrix using t-tests required specifying a nominal significance level, 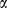. To do so, we plotted the estimated number of potential regulators for several values of 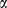. We then chose 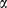 by looking for an ‘elbow’ where the slope of the curve sharply increases; see Figure S2 to see the values selected. After choosing 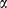, additive ODEs were fit to the time-series and used to rank the potential edges. We set 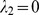 due to the sparsity already introduced using the knockouts and chose 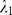 by GCV, searching 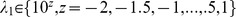 for the 100-node networks and 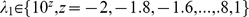 for the 10-node networks.

The results are in Table 7, and include comparisons to teams 315, 304, 256 from the competition for comparison. Again, the results compare favorably particularly considering we made no attempt to optimize the unranked edges eliminated by the prescreening step. We present these comparisons because team 315 was the top performer overall, while teams 304 and 256 were the top performers among those whose methods primarily made use of dynamic models. For this subset of teams, Team 304 was the top performer (fifth overall) on the 100-node networks and Team 256 was the best performer (third overall) on the 50-node networks. Team 304 included the developers of Inferelator, which was a primary component in their larger network reconstruction pipeline [19]. Notably, Team 256 also took a nonparametric approach and utilized ODEs albeit using Bayesian estimation and a different strategy for reconstructing the network from the fitted model [29]. Despite these similarities, our approach offers the advantage of being scalable.

**Table 7:** Results on the DREAM 3 competition data.

	E1		E2		Y1		Y2		Y3	
	PR	ROC	PR	ROC	PR	ROC	PR	ROC	PR	ROC
Team 256, 10-Node	.396	.720	.258	.622	.258	.591	.481	.591	.434	.625
Team 304, 10-Node	.193	.697	.377	.791	.468	.909	.332	.554	.388	.658
Team 315, 10-Node	.710	.928	.713	.912	.897	.949	.541	.747	.627	.714
Additive ODEs, 10-Node	.875	.976	.632	.885	.558	.906	.491	.673	.510	.654
Team 304, 100-Node	.132	.835	.154	.879	.159	.839	.179	.738	.161	.667
Team 315, 100-Node	.694	.948	.806	.960	.493	.915	.469	.856	.433	.783
Additive ODEs, 100-Node	.623	.867	.841	.953	.466	.820	.424	.787	.396	.734

Performance on the *in silico* data for challenge 4 of the DREAM 3 competition [8], [42], [43]. Team 315 was the top performer in the challenge [10], while teams 304 and 256 focused on time series and were among the top performers. Team 304 used Inferelator 1.0 as part of a larger reconstruction pipeline [19]. Team 256 also fit non-parametric ODEs though their approach differed from ours [29]; team 256 did not participate in the 100-node reconstructions due to the complexity of their method. The competition data includes observations for wild-type, knockouts of each gene, heterozygous knockdowns of each gene, and time series under multifactorial perturbations. There are 21 observations on each of the 4 time series for the 10-node networks and the 46 time series for the 100-node networks. Our reconstructions used knockout and time-series data only. PR and ROC values were computed using GeneNetWeaver.

## Discussion

This paper introduces a novel technique, NeRDS, for reconstructing biological networks from time-series data. Unlike other ODE-based approaches which assume a parametric model, we take a nonparametric approach utilizing additive rather than linear approximations. We also introduce a coupling metric that can be used as a general tool for measuring the direct influence of one component on another in nonlinear ODE models. The flexibility of the nonparametric approach allows researchers to proceed with minimal assumptions other than the underlying smoothness inherent to ODE models.

While our approach is flexible, like any nonlinear approach it comes at the price of large data requirements. Specifically, for NeRDS to perform well we require as many time series as network components and that these time series be sufficiently informative. At a minimum, the trajectories of each component must exhibit enough curvature for its regulatory effects to be disambiguated from others on at least some of the time-series experiments. However, in general the number and quality of the time series is much more important than the frequency at which these time series our sampled, provided the sampling is sufficient to capture the system dynamics and maintain some signal amidst the noise. Moreover, time-series data tend to be more readily available then the more informative direct perturbation experiments, such as gene deletion.

Indeed, network reconstruction methods for time series currently lag techniques based on direct perturbations experiments. However their ability to make use of more readily available data is a major advantage, particularly in the early stages of understanding a system— precisely when network reconstruction is most relevant. Given the limitations of current time-series approaches, our method adds to the toolkit for network reconstruction and system identification. No single reconstruction method will be best in all cases. In fact, community network reconstructions that combine information from a variety of algorithms are often superior [44]. Further it expands the class of models available for time-course data to include additive ODEs, thus enriching the collection of methods available for community-based reconstructions.

The flexibility of our method must be balanced against both model and computational complexity. Central to managing these tradeoffs are the tuning parameters: 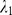 for controlling the smoothness of the additive functions and 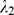 for managing network-level sparsity. Larger choices for these parameters lead to simpler models, smaller choices to additional complexity. For instance, as 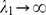 our additive model subsumes a linear models as a special case. The model complexity can also be reduced by limiting the number of interior knots in the basis expansions for the additive functions. While the GCV criterion offers an option for automatic tuning, it tends to err on the side of complexity. Diagnostic plots such as described in the supplement are an invaluable tool in making these selections subjectively. In practice, especially in an exploratory context, we recommend researchers start near the linear case and add additional complexity by decreasing 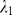 or adding knots as needed. Indeed, early simulation studies on the systems studied in this paper demonstrated that allowing too much complexity (using too many knots or allowing 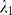 to be too small) significantly reduced performance of the additive ODEs.

Despite the relative complexity of NeRDS we were able to scale to the 100-node networks because the methodology is both modular and easily parallelized. A key reason for the latter is the marginal nature of the reconstruction method. Regression-based approaches such as the current one construct the network by combining the incoming edges selected (ranked) for each node individually. For a network with 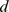 nodes this allows the model fitting to be split into 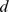 separate tasks. Likewise, the most computationally intensive portion of our methodology— selecting tuning parameters— is trivially parallelized by splitting along each value of the tuning parameter considered in the grid search.

Moreover, by employing basis expansions the nonparametric method allowed us to expand the model class while still only needing to solve linear systems. In addition, since the submodels for all 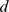 nodes share a single feature space we need to compute only once the matrices defining these linear systems and the decompositions needed to efficiently solve them. Thus while tens of thousands of linear systems were solved in fitting our additive nonparametric models, only a few hundred matrix decompositions were required (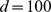 for each value of the smoothing parameter 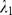 considered).

In relatively small systems, such as the Mouse Embryonic Stem Cell and *Lactocaccus Lactis* systems serving as our primary examples, it appears preferable to fix the sparsity parameter 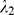 at zero in advance. In contrast, the role of sparsity becomes increasingly important as the number of network nodes grows into the tens and beyond. Moreover, inducing sparsity through 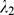 offers the potential to skirt the requirement of as many time series as nodes but at the expense of discovering fewer true edges.

Many of the tradeoffs discussed above are inherent in the problem of reconstructing biological networks and are by no means unique to our method. Generally, there is a continued need for theory to better understand the tradeoffs and how best to manage them. Theory is needed not just for managing tradeoffs within a modeling paradigm, but also for experimental design. Network reconstruction methods based on time series offer two advantages in this regard. First, they rely on the easiest to obtain data and so offering early insight on how to proceed with future experiments. Also, time series methods yield dynamic models useful for estimating the likely information gain from potential experiments.

In order to move toward genome-scale network reconstruction, further work will also be needed to explore how the method presented here fits in with efforts toward data integration. For instance, within the additive framework it is not obvious how to combine multiple time-series datasets not emanating from a single-lab or experimental setup as has been done for linear systems [22]. Determining how to integrate sources of data other than time series, including prior information, network motifs from homologous systems, and steady-state data from perturbation experiments, among others, is a promising direction for further research [6], [45], [46].

By rooting our methodology in statistically-motivated tools we hope in the future to make further theoretical and practical contributions to balancing flexibility and complexity, experimental design for network reconstruction, and data integration.

## Supporting Information

Algorithm S1 **NeRDS Workflow.**
(PDF)

Algorithm S2 **Sparse Backfitting.**
(PDF)

Figure S1
**Diagnostic plots for component 3 (Nanog) in the mouse system.** *Panel A*: Each plot show the normalized observations from one the six simulated experiments as grey dots and the stage-1 smooth as a solid black line. Experiments 4 and 5 appear to carry minimal information for fitting a model to Nanog and are not considered in stage-2 diagnostics. *Panel B:* For each of four relevant experiments, the solid black line is the estimated derivative of Nanog, the dashed red line the unregularized linear fit, and the dot-dash cyan line the additive fit with 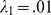, 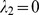. The additive model provides a better fit on the non-dominant experiments.(EPS)

Figure S2
**Plotting the number of potential edges versus 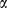.** For the DREAM 3, challenge 4, competition data we used knockout experiments to limit the number of potential regulators. The algorithm used to do this relies on t-tests to determine which gene expression levels in each gene deletion mutant significantly differ from their wild-type expressions. The plots show the number of potential regulators versus the nominal significance level, 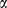, used in these t-tests. We chose 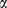 by looking for an ‘elbow’— a location where the slope of the curve sharply increases. The locations indicated by the dashed vertical lines were used for the results presented in Table 6; from left to right these are .03, .02, .015, .025, .015 for the 10-node networks and 
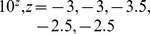
 for the 100-node networks. For the 100-node networks both the number of potential regulators and 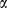 are on the 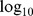 scale. *Top Row:* 10-node networks. *Bottom row:* 100-node networks.(EPS)

Figure S3
**Reconstruction performance on the *Lactocaccus Lactis* network for varied sample size.** Using the experimental setup for *Lactocaccus* described in the Results section of the main paper, we repeated the simulations with reduced sampling densities. The number of observations per time series, 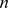, is on the horizontal axis of each plot. Solid, black lines show the performance of the additive ODEs introduced in the paper while dashed, red lines indicate the performance for linear ODEs. The two noise levels, 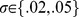, are respectively indicated by round and square symbols. For 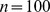 these are the same results presented in Table 1 and Table 2.(EPS)

Figure S4
**Reconstruction performance on the mouse network for varied sample size.** Using the experimental setup for the mouse network described in the Results section, we repeated the simulations with reduced sampling densities indexed by the number observations per time series, 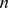, on the horizontal axes. Solid, black lines indicate the performance of the additive ODEs while dashed, red lines show the performance for linear ODEs. The two noise levels, 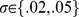, are indicated by round and square symbols, respectively. For 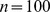 these are the same results presented in Table 3 and Table 4.(EPS)

Text S1 **Diagnostics for tuning parameter selection.**
(PDF)
