# Supplementary material for: Network Reconstruction Using Nonparametric Additive ODE Models
Source: PLoS One. 2014 Apr 14;9(4):e94003. doi: 10.1371/journal.pone.0094003 (PMC3986056; doi:10.1371/journal.pone.0094003)
Supplement: Algorithm S1 — NeRDS Workflow. (PDF) [file pone.0094003.s001.pdf]

Algorithm S1 `NeRDS` Workflow

Stage 1 - Normalize and Smooth the Data
for components $i = 1 : d$ **do**
  input time-course data $(Y_i^r(t_k))_{k=1,r=1}^{n,R}$
  compute $M_i = \max_{k,r}(Y_i^r(t_k))_{k=1,r=1}^{n,R}$
  compute $(\tilde{Y}_i^r(t_k))_{k=1,r=1}^{n,R} = (Y_i^r(t_k)/M_i)_{k=1,r=1}^{n,R}$
  for experiments $r = 1 : R$ **do**
    input normalized time-course data $(\tilde{Y}_i^r(t_k))_{k=1}^{n}$
    compute the trajectory $\hat{x}_i^r(t)$ using smoothing splines
    compute derivative $\hat{\dot{x}}_i^r(t) = \frac{d}{dt}\hat{x}_i^r(t)$
  end for
end for

Stage 2 - Fit an Additive ODE
for components $i = 1 : d$ **do**
  input the derivatives $(\hat{\dot{x}}_i^r(t))_{r=1}^{R}$ as response
  input the trajectories $\{(\hat{x}_j^r(t))_{r=1}^{R}, j = 1, ..., d\}$ as features
  input parameters Λ_1 (smoothing) and Λ_2 (sparsity) to search

  Select Tuning Parameters
  for $\lambda = (\lambda_1, \lambda_2) \in \Lambda_1 \times \Lambda_2$ **do**
    compute $\hat{f}_{i+}(\lambda)$ using Sparse Backfitting [ALGORITHM S2]
    compute $GCV(\lambda)$ [equation (11)]
  end for
  set $\lambda_i \leftarrow \arg\min_{\lambda \in \Lambda_\times \Lambda_2} GCV(\lambda)$

  output $\hat{f}_{i+}(\lambda_i) = \sum_{j=1}^{d} \hat{f}_{ij}(x; \lambda_i)$
end for

Stage 3 - Compute Coupling
for i=1:d **do**
  for j=1:d **do**
    input $\hat{f}_{ij}$
    input $\mathcal{R}_j = \text{range}(\hat{x}_j)$
    compute coupling $\hat{\rho}_{ij}(\hat{f}_{ij}) = \int_{\mathcal{R}_j} [\hat{f}_{ij}(x)]^2 dx / |\mathcal{R}_j|$ [equation (16)]
  end for
end for
