# Supplementary material for: Network Reconstruction Using Nonparametric Additive ODE Models
Source: PLoS One. 2014 Apr 14;9(4):e94003. doi: 10.1371/journal.pone.0094003 (PMC3986056; doi:10.1371/journal.pone.0094003)
Supplement: Algorithm S2 — Sparse Backfitting. (PDF) [file pone.0094003.s002.pdf]

Algorithm S2 Sparse Backfitting

for $i = 1 : d$ **do**
  input Concatenated derivative estimates: $\hat{\dot{\boldsymbol{x}}}_i = (\hat{\dot{x}}_i^1(t_1), ..., \hat{\dot{x}}_i^R(t_n))'$.
  input Concatenated trajectory estimates: $\hat{\boldsymbol{x}}_j = (\hat{x}_j^1(t_1), ..., \hat{x}_j^R(t_n))'$,
    $j = 1, ..., d$.
  input Smoothing parameter λ_{1i} and sparsity parameter λ_{2i}.
  initialize $\boldsymbol{f}_{ij} \leftarrow 0 \in \mathbb{R}^N$, for $j = 1, ..., d$, where $N = nR$.
  compute $\hat{\alpha}_i \leftarrow N^{-1} \sum_{k=1}^{N} \hat{\dot{\boldsymbol{x}}}_{ik}$.
  repeat
    Store starting values $\boldsymbol{f}_i^\star \leftarrow \boldsymbol{f}_i$.
    for $j = 1 : d$ **do**
      Compute residuals $\boldsymbol{r} \leftarrow \hat{\dot{x}}_i - \hat{\alpha}_i - \sum_{\ell \neq j} \boldsymbol{f}_{i\ell}(\hat{\boldsymbol{x}}_\ell)$
      Smooth $\boldsymbol{f}_{ij} \leftarrow S_j(\lambda_{1i})\boldsymbol{r}$
      Estimate Norm $s^2 \leftarrow \left(N^{-1} \sum_{k=1}^{N} \boldsymbol{f}_{ijk}^2\right)^{\frac{1}{2}}$
      Soft Threshold $\boldsymbol{f}_{ij} \leftarrow (1 - \lambda_{2i}/s^2)_+ \boldsymbol{f}_{ij}$
      Center $\boldsymbol{f}_{ij} \leftarrow \boldsymbol{f}_{ij} - \bar{\boldsymbol{f}}_{ij}$
    end for
  until *Convergence* $\sup_{j=1,...,d} \sup_{k=1,...,N} |\boldsymbol{f}_{ijk} - \boldsymbol{f}_{ijk}^\star| < \epsilon$.
end for
