# Supplementary material for: Network Reconstruction Using Nonparametric Additive ODE Models
Source: PLoS One. 2014 Apr 14;9(4):e94003. doi: 10.1371/journal.pone.0094003 (PMC3986056; doi:10.1371/journal.pone.0094003)
Supplement: Text S1 — Diagnostics for tuning parameter selection. (PDF) [file pone.0094003.s007.pdf]

## TEXT S1. DIAGNOSTICS FOR TUNING PARAMETER SELECTION.

In order to fit an additive ODE to a collection of time series using `NeRDS`, four types of tuning parameters need to be selected. These are: (1) $\lambda_0$ controlling smoothness in the first stage; (2) $\lambda_1$ and the number of knots, controlling smoothness of the additive functions in stage 2; and (3) $\lambda_2$ controlling sparsity of the additive components in stage 2.

In the first stage, smoothing splines are fit to each component $(i = 1, ..., d)$ of each time series $(r = 1, ..., R)$ yielding estimates of the trajectories $\hat{x}_i^r(\cdot)$ and derivatives $\hat{\dot{x}}_i^r(\cdot)$. The smoothness of these trajectories are determined by smoothing parameters $\lambda_{i0}^r$ and can be efficiently selected by GCV (which we use) or even standard cross validation. Once these estimates are obtained, it is useful to overlay plots of the observations $Y_i^r(t_k)$ and the estimated trajectories $\hat{x}_i^r(t)$ against time for each experiment $r$. See Figure S1, panel A for an example. If the any of the estimated trajectories appear jagged, they may be over fit and $\lambda_{i0}^r$ should be adjusted upwards to give a smoother estimate. This is of particular importance when the sampling density is low relative to the amount of noise.

For each submodel $(i = 1, ..., d)$ in the second stage the smoothness of the additive components $(j = 1, ..., d)$ is determined by $\lambda_{i2}$ and the number of knots. As presented in the paper, this smoothing parameter can also be selected by GCV though this will often lead to overfitting due the noise in the derivatives $\hat{\dot{x}}_i^r(\cdot)$. As before, overlaying plots of the estimated derivatives and select additive fits versus time for each experiment $r$ is useful for appropriately balancing flexibility and complexity.

As an example Figure S1 shows such plots for submodel 3 (Nanog) from the mouse system. Based on the smooths from the first stage (panel A), experiments $r = 4$ and $r = 5$ appear relatively unimportant due to the small rate of change in the trajectories of Nanog. For the remaning four experiments, $r = 1, 2, 3, 6$ we overlay plots of the estimate derivatives (black, solid), the linear fit (red, dashed), and an additive fit with 4 knots, $\lambda_2 = 0$, and $\lambda_1 = .01$ (cyan, dot-dash).

The additive model provides a better fit than the linear model while still being smoother than the estimated derivative it approximates indicating an appropriate balance between complexity and flexibility. If the fit is inadequate additional flexibility can be added by reducing $\lambda_1$ or incorporating additional knots. In contrast, if a similar fit can be achieved with larger $\lambda_1$ or fewer knots the simpler model should be preferred. In our simulations with the mouse system, we chose $\lambda_1$ by GCV but set the lowest value in the line search to .01 based on these plots. This served to prevent overfitting while allowing GCV to choose still less complexity when warranted by the data.

The final tuning parameter to be set is $\lambda_2$ controlling sparsity of the additive fits in stage 2. In settings where $d$ is small, our experience in simulations has been that setting $\lambda_2 = 0$ or very small is best. However, for larger $d$, like in the DREAM 3 100-node networks, choosing $\lambda_2 > 0$ not only induce sparsity in the fitted submodels but also greatly speeds computation. When employing prescreening as we did with the DREAM 3 competition data, it may be possible to choose $\lambda_2 = 0$ as long as the number of potential regulators in each submodel is small. For $\lambda_2$ large enough, the null model with all the additive components identically zero will be returned. In practice, one can examine the same plots as for $\lambda_1$ to determine if a particular value of the sparsity parameter $\lambda_2$ allows adequate flexibility. One may wish to find a $\lambda_2$ leading to the null model and then progressively decrease until either: (1) adequate fit is seen in diagnostic plots; (2) the number of

non-zero components is the additive fits are sufficiently large relative to prior knowledge on the approximate in-degree; (3) the computational burden becomes too much to decrease further. GCV remains an option for jointly determining $\lambda_1$ and $\lambda_2$ when looking at plots is impractical.

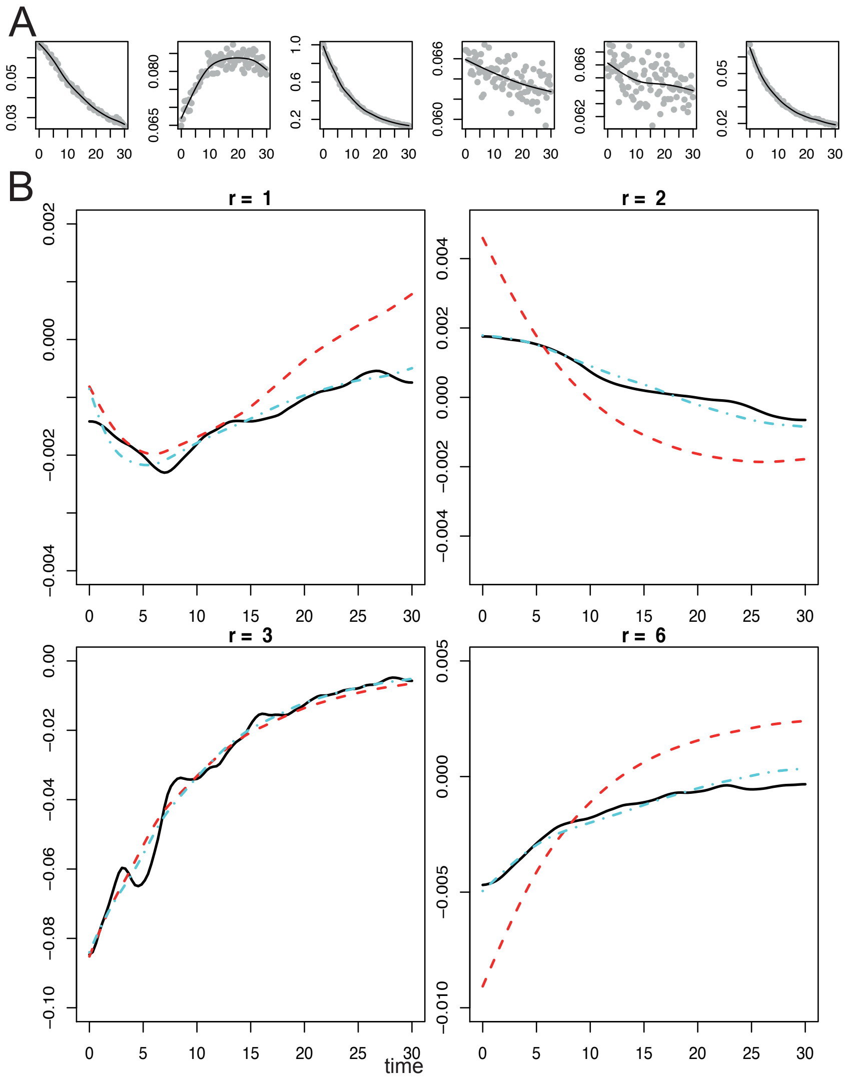


Figure S1. **Diagnostic plots for component 3 (Nanog) in the mouse system.** *Panel A*: Each plot show the normalized observations from one the six simulated experiments as grey dots and the stage-1 smooth as a solid black line. Experiments 4 and 5 appear to carry minimal information for fitting a model to Nanog and are not considered in stage-2 diagnostics. *Panel B:* For each of four relevant experiments, the solid black line is the estimated derivative of Nanog, the dashed red line the unregularized linear fit, and the dot-dash cyan line the additive fit with $\lambda_1 = .01$, $\lambda_2 = 0$. The additive model provides a better fit on the non-dominant experiments.
